# Supplementary material for: Is Short-Term Exposure to PM2.5 Relevant to Childhood Kawasaki Disease?
Source: Int J Environ Res Public Health. 2021 Jan 21;18(3):924. doi: 10.3390/ijerph18030924 (PMC7908497; doi:10.3390/ijerph18030924)
Supplement: Supplementary file 1 [file ijerph-18-00924-s001.pdf]

Supplementary files

Title: Is short-term exposure to $PM_{2.5}$ relevant to childhood Kawasaki disease?

Jongmin Oh[1*], Ji Hyen Lee[2*], Eunji Kim[1,3], Soontae Kim[4], Hae Soon Kim[2†], Eunhee Ha[1,3†]

1. Department of Occupational and Environmental Medicine, Ewha Womans University College of Medicine, Seoul, the Republic of Korea.
2. Department of Pediatrics, Ewha Womans University College of Medicine, Seoul, the Republic of Korea
3. System Health & Engineering Major in Graduate School (BK21 Plus Program), Ewha Womans University, Seoul, the Republic of Korea.
4. Department of Environmental and Safety Engineering Ajou University, Suwon, the Republic of Korea.

Co-first author: * Jongmin Oh and Ji Hyen Lee contributed equally to this study.

Co-Corresponding author: † Professor Eunhee Ha, MD, PhD

Department of Occupational and Environmental Medicine, Ewha Womans University College of Medicine

260, Gonghang-daero, Gangseo-gu, Seoul 07804, Republic of Korea

E-mail: eunheeha@ewha.ac.kr

Co-Corresponding author: † Professor Hae Soon Kim

Department of Pediatrics, Ewha Womans University College of Medicine

260, Gonghang-daero, Gangseo-gu, Seoul 07804, Republic of Korea

E-mail: hyesk@ewha.ac.kr

Supplementary files

Table S1. Frequency of residential areas (administrative districts) by Kawasaki disease patients.

Regions	districts	Kawasaki disease cases
Seoul	Gangseo-gu	268
Seoul	Yangcheon-gu	215
Seoul	Guro-gu	76
Gyeonggi	Gwangmyeong-si	50
Seoul	Yeongdeungpo-gu	37
Gyeonggi	Gimpo-si	21
Gyeonggi	Bucheon-si	21
Incheon	Seo-gu	11
Seoul	Eunpyeong-gu	10
Seoul	Mapo-gu	8

Table S2. Summary statistics for daily air pollutants measured by a monitoring station during the study period of 2006-2016.

	Mean	SD	Median	IQR
PM_{10} (μg/m^3)	50.669	32.270	44.138	30.847
SO_2 (ppm)	0.006	0.002	0.006	0.003
NO_2 (ppm)	0.033	0.014	0.031	0.019
CO (ppm)	0.543	0.483	0.256	0.275
O_3 (ppm)	0.022	0.012	0.020	0.017

Abbreviations: SD, standard deviation; IQR, interquartile range.

Table S3. Spearman's correlation matrix between daily air pollutants during the study period (2006–2016)

	**$PM_{2.5}$**	**PM_{10}**	**SO_2**	**NO_2**	**CO**	**O_3**
**$PM_{2.5}$**	1.00	0.92	0.51	0.52	0.56	-0.07
**PM_{10}**		1.00	0.46	0.44	0.50	-0.03
**SO_2**			1.00	0.54	0.51	-0.16
**NO_2**				1.00	0.70	-0.45
**CO**					1.00	-0.42
**O_3**						1.00

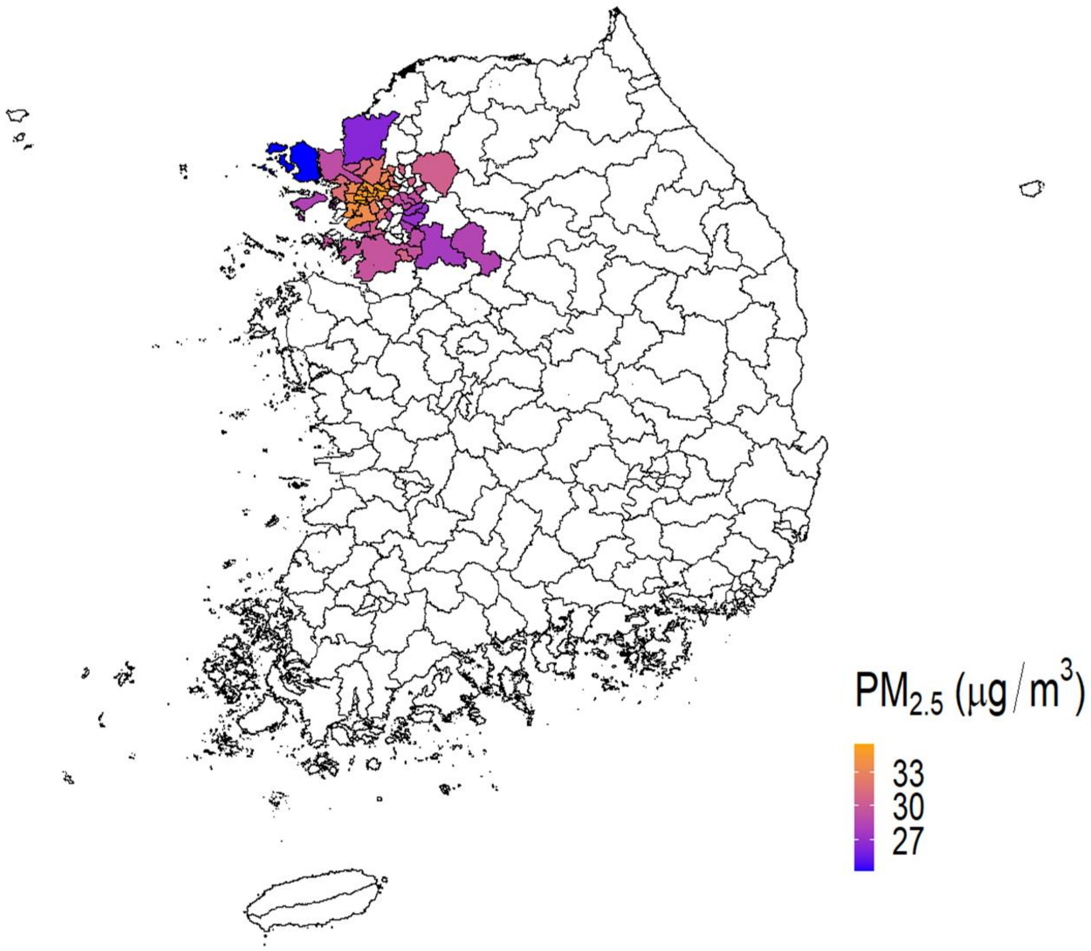

Figure S1. Average annual exposure concentration of $PM_{2.5}$ in each administrative district during 2006-2016

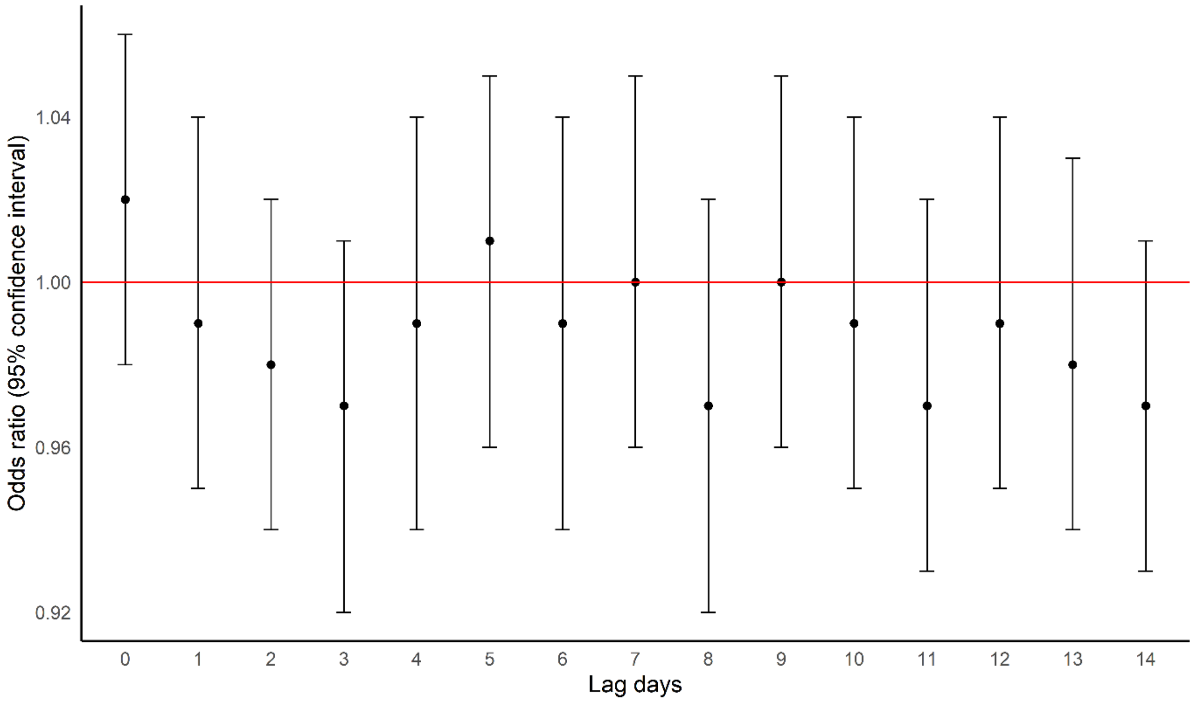

Figure S2. Single-lag effect from the current day (lag 0) of exposure to $PM_{2.5}$ and children's KD hospitalizations to two weeks ago. The red line indicates the borderline. All models were adjusted for the daily mean temperature and humidity. The odds ratio was calculated per 10-unit increase.
